# Antibacterial potential of silver and zinc loaded plasma-electrolytic oxidation coatings for dental titanium implants

**DOI:** 10.1186/s40729-025-00595-w

**Published:** 2025-02-17

**Authors:** Sabawun Paiwand, Sogand Schäfer, Alexander Kopp, Thomas Beikler, Imke Fiedler, Martin Gosau, Sandra Fuest, Ralf Smeets

**Affiliations:** 1 Department of Oral and Maxillofacial Surgery, University Medical Center Hamburg-Eppendorf, Martinistrasse 52, 20246 Hamburg, Germany; 2 Department of Neurosurgery, University Medical Center Schleswig-Holstein, Campus Kiel, Arnold-Heller-Strasse 3, 24105 Kiel, Germany; 3 Department of Oral and Maxillofacial Surgery, Devision of Regenerative Orofacial Medicine, University Medical Center Hamburg-Eppendorf, Martinistrasse 52, 20246 Hamburg, Germany; 4 Division of Plastic and Reconstructive Surgery, Department of Surgery, Children’s Hospital of Philadelphia, 3401 Civic Center Blvd., Philadelphia, PA 19104 USA; 5 Meotec GmbH, Philipsstraße 8, 52068 Aachen, Germany; 6 Department of Periodontics, Preventive and Restorative Dentistry, University Medical Center Hamburg-Eppendorf, Martinistrasse 52, 20246 Hamburg, Germany; 7 Department of Osteology and Biomechanics, University Medical Center Hamburg-Eppendorf, Martinistrasse 52, 20246 Hamburg, Germany

**Keywords:** Antibacterial, Dental implants, Zinc, Silver, Plasma-electrolytic oxidation, Peri-implantitis

## Abstract

Peri-implantitis is known as an inflammatory condition affecting the soft and hard tissue around dental implants. A promising strategy to prevent these conditions is the use of antibacterial implants. This study aimed to evaluate the antibacterial potential of titanium (Ti) dental implants modified using plasma-electrolytic oxidation (PEO). The modified surfaces were subsequently loaded with silver (Ag) (*n* = 6) and zinc (Zn) (*n* = 6) ions and compared to unloaded Ti specimens (*n* = 6), with untreated specimens serving as controls. The specimens (each *n* = 5) were incubated in a culture medium containing a mixture of specific anaerobic bacterial strains. Scanning electron microscopy (SEM) was used to visualize the bacterial biofilm on each specimen. In addition, total bacterial deoxxyribonucleic acid (DNA) and the number of viable bacteria were determined using quantitative real-time polymerase chain reaction (qrt-PCR) and colony forming unit analysis (CFU), respectively. The results of the CFU analysis showed a 2 log (99%) reduction in viable bacteria in the samples loaded with Ag and Zn compared to the unloaded control group (p < 0.05). Moreover, significantly lower bacterial DNA counts were detected with a 5 log reduction (99.999%) in the Ag and Zn samples compared to the positive control group (bacterial mixed culture solution, p < 0.05). Therefore, it was considered that Ag and Zn loaded Ti implants may be a promising addition to current approaches to enable advanced antibacterial dental implants. However, further studies should be conducted to evaluate the in vivo cytocompatibility of the developed specimens.

## Background

Since the 1960s, titanium (Ti) implants made of commercially pure Ti or certain Ti alloys (Ti-6Al-4 V, Ti-12Zr, and many others) have been used in dentistry due to their exceptional properties, such as biocompatibility, high corrosion, and fatigue resistance as well as a high strength-to-weight ratio [1]. Despite the widespread use of Ti implants, there are still many concerns regarding the occurrence of infections at the implant site. The oral cavity is a moist and warm environment that provides ideal conditions for the growth of commensal and pathogenic bacteria such as *Porphyromonas gingivalis*, *Centipeda periodontii*, *Parvimonas micra*, *Eubacterium nodatum*, *Filifactor alocis*, *Parascardovia denticolens*, *Eubacterium brachy*, *Prevotella intermedia*, *Slackia exigua*, *Fusobacterium nucleatum*, and *Eubacterium saphenum* [2]. These bacteria can adhere to the surface of teeth or inserted implants and form a biofilm called plaque, which is the main cause of peri-implantitis. Peri-implantitis is an inflammatory disease of the soft and hard tissue surrounding the implant that can lead to implant failure, even if the implant and alveolar bone exhibit sufficient osseointegration. To prevent peri-implantitis, several methods have been used to reduce bacterial growth and colonization at the implant site [3–5]. One of these approaches is the biofunctionalization of surface implants with bactericidal agents [6]. Dissolved Ti ions have shown an antibacterial effect, however this was not sufficient to prevent local infection by the bacterial flora of the oral cavity [7]. To increase the antibacterial effect, Ti implants can be loaded with bactericidal agents instead [8]. The choice of antibacterial agents is critical in this context. To date, various bactericidal agents such as inorganic substances, e.g., fluoride [9, 10], chlorhexidine [11], gentamicin [12], cephalothin [13], amoxicillin [14] and organic substances, e.g., copper [15], gold [16], zinc (Zn) [17] and silver (Ag) [18] have been applied to Ti-based implants to provide antibacterial properties. Ag ions are widely known for their specific anti-inflammatory and anti-bacterial properties at low concentrations [19], making them a cost-effective option with low cytotoxic effect on the immune system [20]. These properties have made Ag ions valuable for various biomedical applications, including antibacterial coatings for implants, wound dressings and meshes for joint replacements [21, 22]. In many studies, Ag ions have been increasingly used as surface modification agents for dental implants to gain bactericidal properties [18, 23, 24]. However, the biocompatibility of Ag ions is controversial and with increasing concentrations they exhibit toxicity in some cell lines [25, 26].

Zn ions, as well as Ag ions, are known to be trace elements involved in various cellular processes, such as cell division, enzyme activity and DNA synthesis [27]. These metal ions have five times less toxicity to mesenchymal stem cells compared to Ag ions [28]. In addition, they can stimulate bone tissue formation, enhance osteogenic function in osteoblasts, increase alkaline phosphatase activity, and promote collagen and protein synthesis [29, 30]. Zn ions have been shown to have an antibacterial effect due to a significant enhancement of oxidative stress in bacteria cells. Consequentially, many studies have loaded Zn ions onto the surfaces of bioimplants to achieve an antibacterial effect [31–33].

Further to antibacterial loading, stimulation of rapid osteointegration immediately after implantation is another potential technique to reduce the risk of infection [34]. In this context, special surface treatments of dental implants create a porous surface structure that triggers the formation of de novo bone and leads to complete osseointegration. This timely integration simultaneously prevents infection by reducing bacterial adhesion [35].

In addition to sandblasted and etched surfaces, plasma electrolytic oxidation (PEO) also known as micro-arc oxidation is one of the most important approaches to produce biofunctionality for Ti based dental implants [36]. PEO forms a porous surface layer on Ti implants that promotes osseointegration by increasing osteoblast proliferation and collagen synthesis threefold compared to untreated Ti implants [37]. This layer increases the surface area available for implant biofunctionalization and promotes the release of incorporated ions [38]. Through this method, different ions can be incorporated into the electrolyte solution at different concentrations to provide the desired biological responses on the surface of the implants. So far, ions such as Ca, P, Mg, Si, Zn and Mn have been applied to Ti implants [39, 40]. This study combines two critical approaches to improve dental implant outcomes through surface modification and providing antibacterial properties to prevent peri-implantitis. Accordingly, PEO surface modification in a modified electrolyte system was used to incorporate Ag and Zn ions onto the surface of Ti implants. Ti + PEO(+ Ag) and Ti + PEO(+ Zn) were fabricated as multifunctional implant specimens and their antibacterial potential was investigated for dental applications.

## Materials and methods

### Specimen production

To simulate the as-manufactured condition of dental implants made of commercially pure Ti, rods of Ti grade 4, according to ASTM F67, were turned down to dimensions of D 18 mm × L 2 mm on a SWISS lathe (Swiss GT13, Tornos Technologies Deutschland GmbH, Pforzheim, Germany). Following degreasing and cleaning, 18 Ti specimens’ surfaces were modified by PEO using three different electrolyte variations. Three different PEO surfaces were prepared using distinctive combinations of electrolyte components based on commercially used IROX1^®^ electrolyte with (Ti + PEO(+ Ag) and Ti + PEO(+ Zn), each n = 6) or without (Ti + PEO, n = 6) addition of either Ag- or Zn-containing chemical compounds at 10 g/L. PEO was achieved using a pulsed rectifier set (Meotec M-PEO A1, Meotec GmbH, Aachen, Germany). Positive and negative pulsed galvanostatic currents of 0.4–0.9 A with voltages from 0–300 V were applied using two feed cables connected to an electrochemical cell consisting of the Ti specimen acting as the anode and the cool-jacketed stainless-steel container acting as the cathode. The pulse frequency was set to 10 Hz while the discs were treated for up to 10 min. After processing, all samples were rinsed with distilled water in an ultrasonic bath for 15 min and dried on a sterile cloth at room temperature.

### Cytocompatibility assessment

#### Cell culture

L929 mouse fibroblasts (LGC Standards, Wesel, Germany) were cultured in Minimum Essential Medium supplemented with 10% fetal bovine serum, penicillin/streptomycin (100 U/ml each) (all from Life Technologies, Carlsbad, USA) and L glutamine (Sigma–Aldrich, St. Louis, USA) in 12 well plates to a final concentration of 4 mM at 37 °C, 5% CO2 and 95% humidity. Cells that had reached 80% confluence were passaged.

#### Reference materials

Reference Material A (RM-A, Hatano Research Institute, Food and Drug Safety Center, Japan) was used as a toxic control. Tissue culture coverslips (TCC, Sarstedt, Nürmbrecht, Germany) were used as a non-toxic control.

#### Live-dead staining assay

Cytocompatibility was assessed using a live-dead staining assay with L929 cells as described above [41]. Briefly, samples were seeded directly with L929 cells in a cell culture medium at a surface-to-volume ratio of 5.65 cm^2^/ml in wells of a 12-well plate. After incubation for one day under cell culture conditions, 60 µl per ml propidium iodide stock solution (50 µg/ml in PBS) and 500 µl per ml medium fresh fluorescein diacetate (FDA) working solution (20 µg/ml in PBS from 5 mg/ml FDA in acetone stock solution) were added to each well. Following a brief incubation of 3 min at room temperature, the specimens were rinsed in PBS and immediately visualized with an upright fluorescence microscope (Nikon ECLIPSE Ti–S/L100, Nikon, Düsseldorf, Germany).

#### Antibacterial activity

All specimens were autoclaved to create sterile conditions (AUTOCLAVE SYSTEC V-40, Systec GmbH, Linden, Germany) and then incubated for 24 h in an artificial saliva consisting of a-amylase 1 mg/ml, mucin 0.85 mg/ml, and bovine serum albumin (BSA) 0.4 mg/ml.

Cultivation of bacterial strains and following treatment of the specimens was performed under anaerobic conditions at 37 °C in a Whitley A35 Workstation (Whitley A35 Workstation Don Whitley Scientific, Bingley, United Kingdom). Anaerobic stains of early colonizing *Streptococcus mutans* (Streptococcus mutans, DSM 20523, Deutsche Sammlung von Mikroorganismen und Zellkuturen GmbH, Leibnitz, Germany), moderately colonizing *Actinomyces naeslundii* (*Actinomyces naeslundii*, DSM 17233, ibid.), *Fusobacterium nucleatum* (*Fusobacterium nucleatum,* DSM 15643, ibid.), and late colonizing *Porphyromonas gingivalis* (*Porphyromonas gingivalis*, DSM 20709, ibid.) were separately cultivated on blood agar plates. Afterwards, each strain was transferred into a bacterial culture medium (CDC Anaerobe 5% Sheep Blood), and after sufficient observable growth (usually after 2–5 days), 1 ml of each strain was mixed into 20 ml CDC medium. The optical density of the mixed bacterial culture was 0.1. Five specimens (*n* = 5) of each implant variant, Ti + PEO, Ti + PEO(+ Ag), and Ti + PEO(+ Zn), were placed on 6-well plates. Each well was previously filled with 1.5 ml of mixed bacterial culture suspension. One specimen of each served as a negative control and was therefore incubated in DMPBS only. The prepared mixed bacterial culture was used as a positive control. After an incubation period of 48 h, specimens were placed separately into sterile 6-well plates previously filled with 1.5 ml DMPBS, vortexed, and a total of 100 µl of specimens were collected from each well for analysis of total bacterial count and number of viable bacteria. Viable bacterial count was determined by inoculating blood agar plates with 50 µl specimen in dilutions of 1:100, 1:1000, 1:10.000 and the Colony Forming Units (CFU) was measured. Total bacterial DNA count was determined through Polymerase Chain Reaction (PCR) by processing 50 µl specimen with DNA Isolation Kit (innuPREP DNA Isolation Kit, Analytik Jena AG, Jena, Germany) and quantifying the amount of DNA with qrt-PCR (CFX96 Touch Real-Time PCR Detection System, Bio-Rad Laboratories, Berkeley, California, USA) utilizing a universal eubacterial 16S-rRNA primer (had1GACTCCTACGGGAGGCAGCAGT, E1115RAGGGTTGCGCTCGTTGCGGG) and additional specific primers (Table 1).

**Table 1:** Specific primer sequences for qrt-PCR and references of their applicability

Organism	Primer	Primer sequence	Reference of primer applicability
*Porphyromonas gingivalis*	CA-PG-F/R	AGGCAGCTTGCCATACTGCG ACTGTTAGCAACTACCGATGT	[42]
*Streptococcus mutans*	MKD-FV/RV	GGCACCACAACATTGGGAAGCTCAG GGAATGGCCGCTAAGTCAACAGG	[43]
*Actinomyces species*	ACT-174-F ACT-281-R	GGTCTCTGGGCCGTTACTGA GRCCCCCCACACCTAGTG	[44]
*Fusobacterium nucleatum*	CA-FN-F/R	AGAGTTTGATCCTGGCTCAG GTCATCGTGCACACAGAATTGCT	[42]

#### Scanning electron microscopy

The surface morphology of the incubated specimens, as well as the elemental composition were analyzed using SEM (Crossbeam 340, Zeiss, Oberkochen, Germany) and energy dispersive X-ray spectroscopy (EDS) (Octane Plus, EDAX LLC, Pleasanton, California, USA). The specimens were then mounted on a specimen holder and sputtered with gold to increase conductivity. Imaging was performed in secondary electron (SE) mode at an acceleration voltage of 5 kV, a working distance of 5 mm, and at magnifications ranging from 3750 X to 20,000 X.

#### Statistical analysis

Statistical analysis of the antibacterial assays was conducted using SAS 7.4 (SAS Institute Inc., Cary, North Carolina, USA).

Descriptive analyses involved the calculation of mean values and standard deviations. For inferential analyses, a generalized linear model (GLM) with an assumed exponential distribution was applied. Depending on the research question, either the qRT-PCR values or the CFU counts were used as the dependent variable.

Specimens were included in the model as fixed effects. Post-hoc comparisons were based on marginal means, with p-values adjusted for multiple testing within the model using the Bonferroni correction. Adjustments for multiple testing were not performed between models.

A significance level of α = 0.05 was defined, and p-values below this threshold were considered to be statistically significant.

## Results

### Surface morphology and elemental composition

**Fig. 1:**
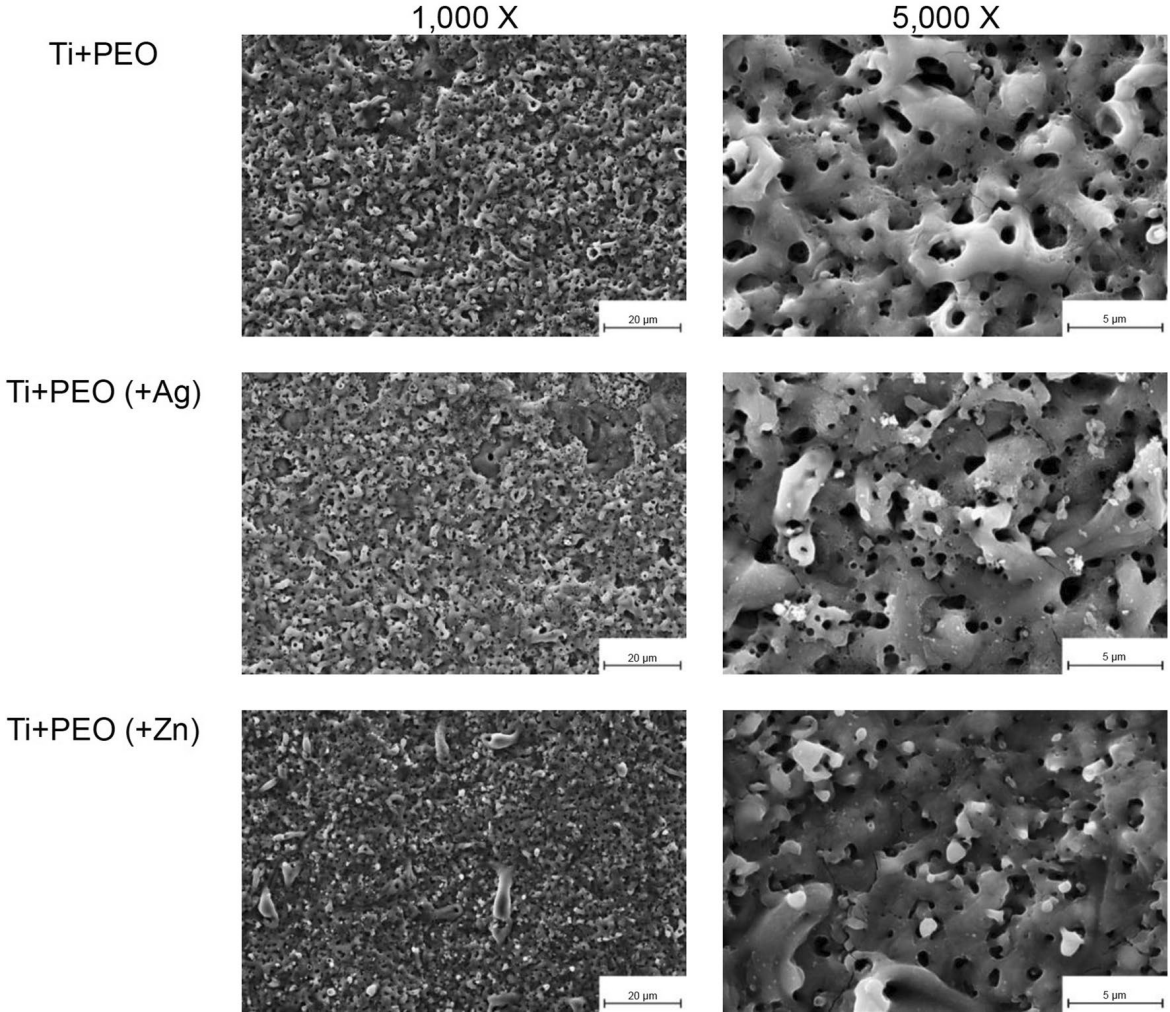
SEM images of Ti + PEO, Ti + PEO(+ Ag), Ti + PEO(+ Zn) specimens with magnifications of 1,000 x and 5,000 x

**Fig. 2:**
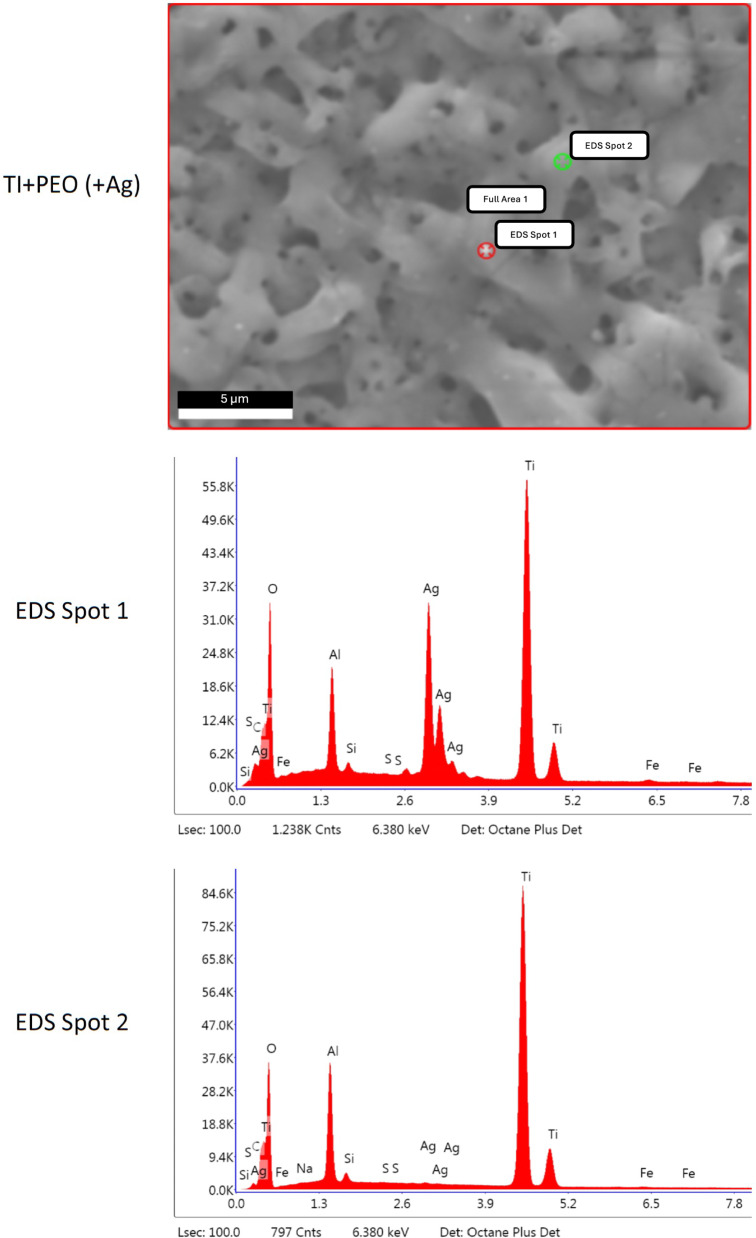
EDS spot analysis of Ti + PEO(+ Ag) specimens, while spot 1 is a particulate area and spot 2 is a non-particulate area

**Fig. 3:**
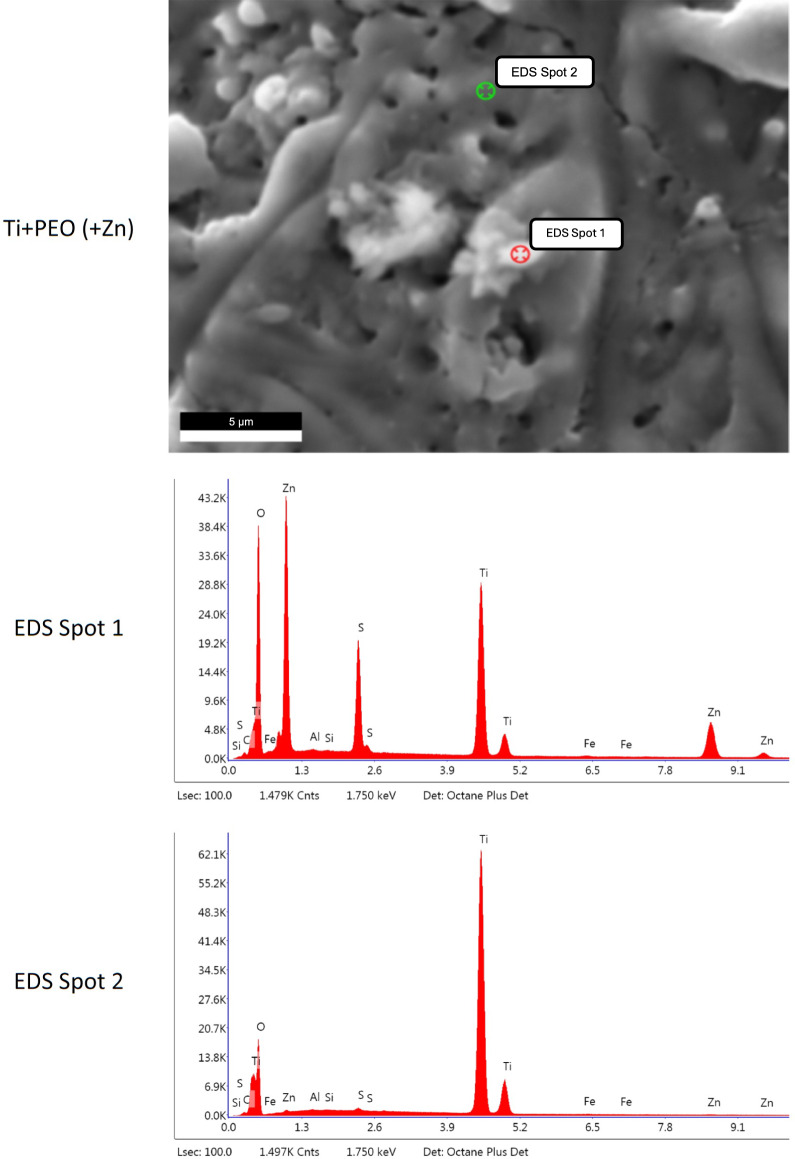
EDS spot analysis of Ti + PEO(+ Zn) specimens, while spot 1 is a particulate area and spot 2 is a non-particulate area

**Fig. 4:**
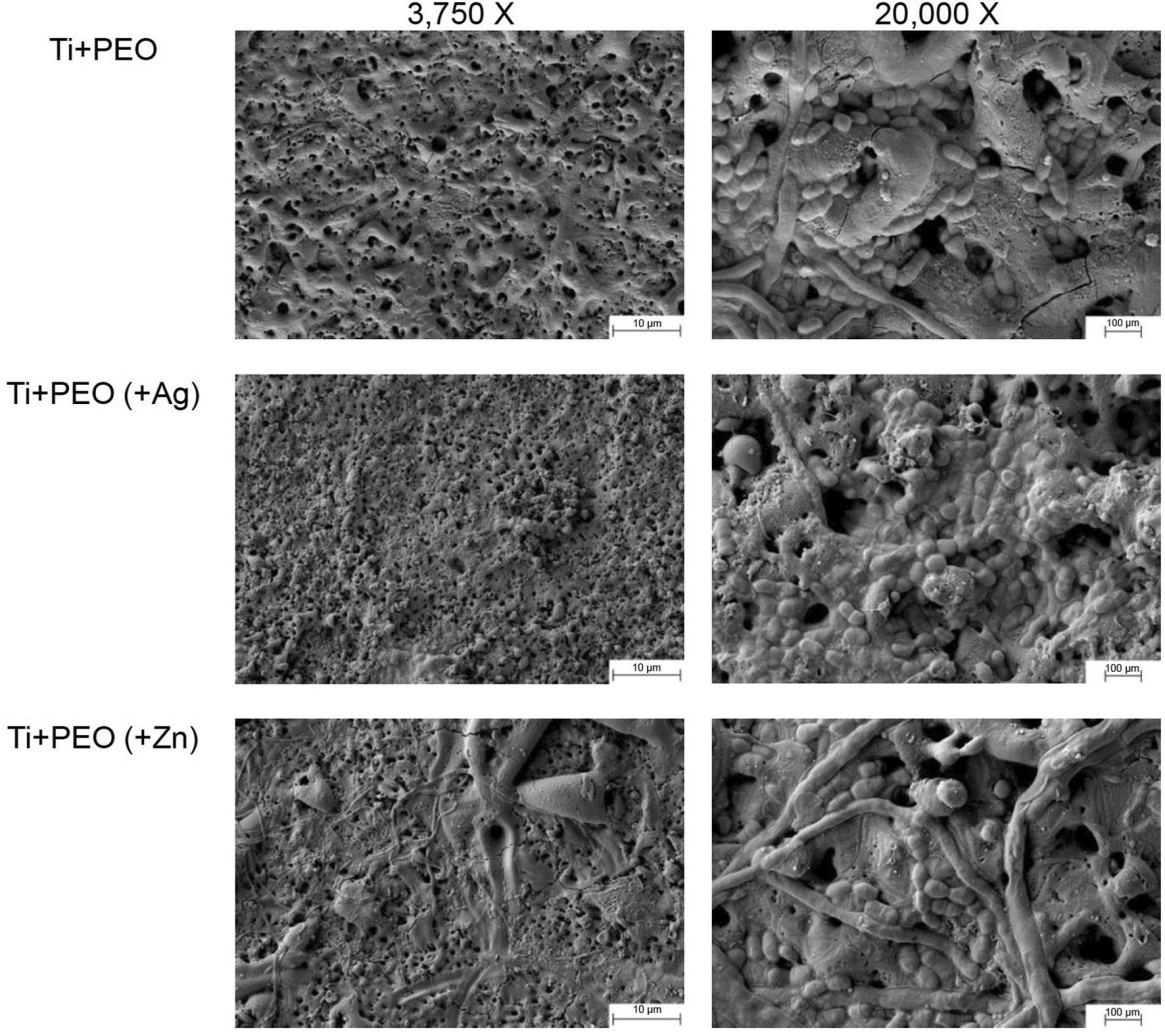
SEM images of Ti + PEO, Ti + PEO(+ Ag), Ti + PEO(+ Zn) specimens after bacterial incubation at magnifications of 3,750 x and 20,000 x

The porous topography of all PEO-treated samples was visualized with SEM (Fig. 1). The additional EDS analysis confirmed the successful loading of the PEO specimens with either Ag or Zn. Spot analyses revealed that Ag was present at high levels in the particulate area (EDS Spot 1, Fig. 2) but was detected without a peak in the non-particulate area (EDS Spot 2, Fig. 2). Similarly, the presence of Zn in a particulate form on the surface was demonstrated (Fig. 3). After incubating the specimens with a mixed bacterial culture, different bacterial colonies were morphologically visible on all specimens, confirming the incubation method used (Fig. 4).

### Cytocompatibility

**Fig. 5:**
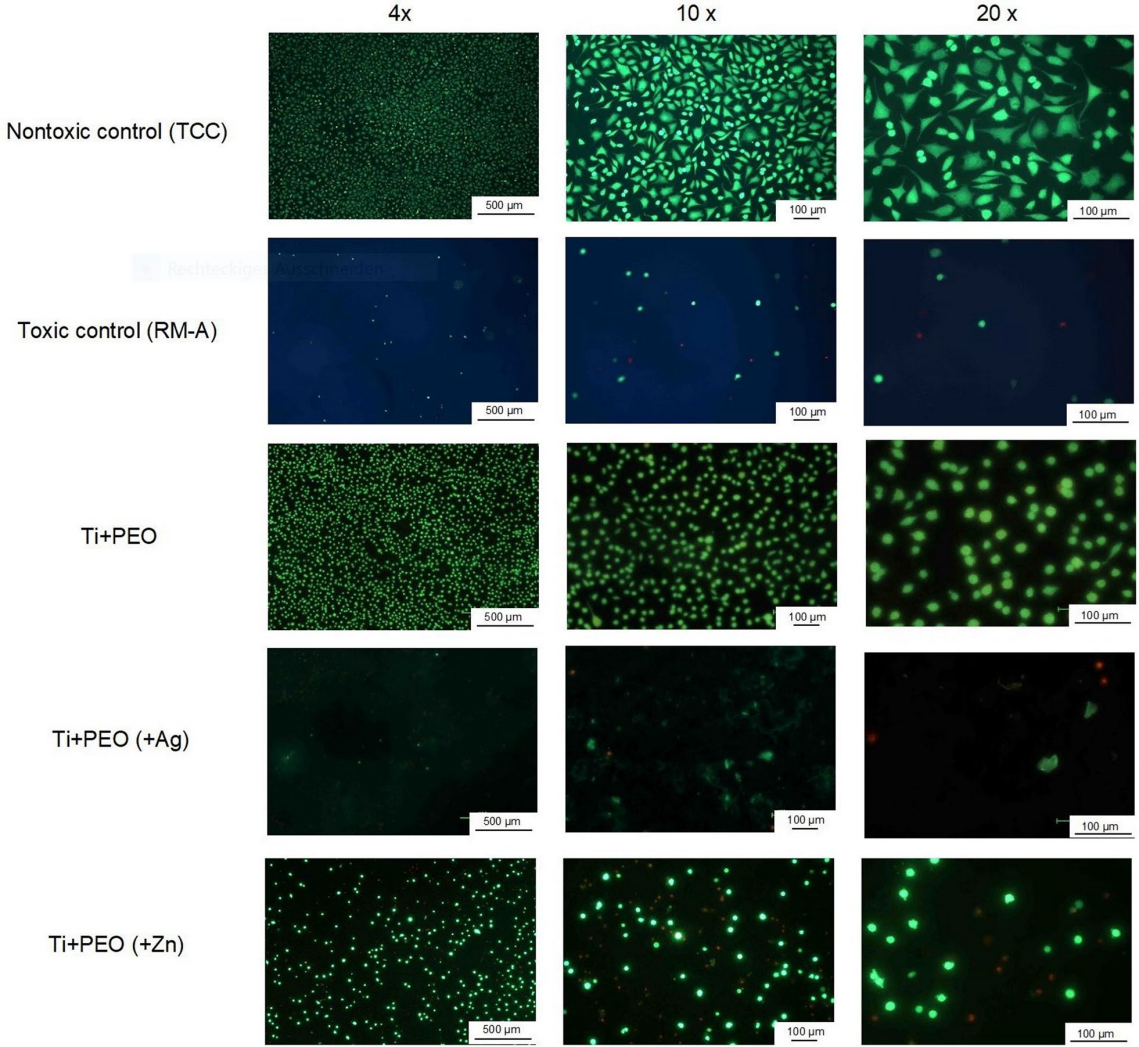
Live-dead staining assay with L929 fibroblasts seeded on PEO-treated Ti samples with Ag and Zn (Ti + PEO(+ Ag) and Ti + PEO(+ Zn) and without the addition of metal ions acting as antibacterial agents (Ti + PEO). Positive control group: Nontoxic control (TCC)

Live-dead staining revealed many green, fluorescein diacetate (FDA) positive vital cells on the Ti + PEO and Ti + PEO(+ Zn) specimens. On the Ti + PEO(+ Ag) specimens, the number of living cells was noticeably reduced compared to the other specimens, however, propidium iodide (PI) positive cells were absent as well (Fig. 5). Furthermore, spindle-shaped cells with the typical morphological characteristics of well spread fibroblasts were detected on the Ti + PEO and most Ti + PEO(+ Zn) samples. In contrast, fibroblast cells on the Ag-containing samples (when detectable) and to a lesser extent on the Zn-containing samples, showed a rounded morphology. Overall, these results indicate no cytotoxicity for all Ti + PEO specimens, but slightly reduced cell counts and considerably reduced cell viability for Ag and Zn-loaded materials.

### Antibacterial activity

#### Viable bacterial count

**Fig. 6:**
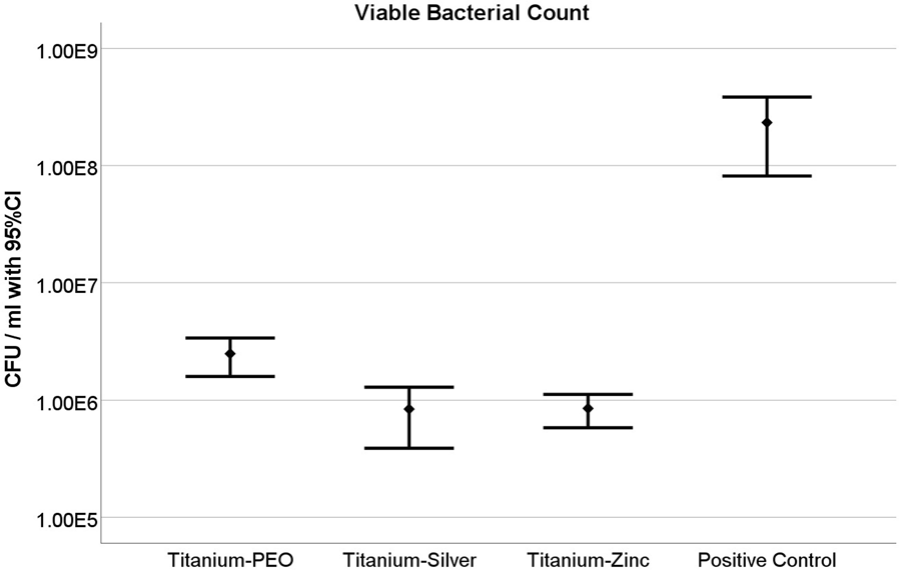
Colony Forming Unit/mL of Ti + PEO, Ti + PEO(+ Ag) and Ti + PEO(+ Zn) after 48 h of specimen incubation on plated mixed bacterial culture solution. Mean values with 95% confidence interval are given in logarithmic scale unit. Results indicate viable bacterial count. *N* = 5/implant variant; negative control (*n* = 3); positive control (*n* = 3). *p < 0.05

In relation to the viable bacterial count, the positive control shows a reference value of M = 2.33E + 08 CFU with a standard deviation of SD = 6.110E + 07. In direct comparison, Ti + PEO achieves a value of M = 2.50E + 06 CFU (SD = 7.271E + 05). At M = 8.41E + 05 CFU (SD = 3.640E + 05), the material Ti + PEO(+ Ag) achieves a marginally higher value than Ti + PEO. Ti + PEO(+ Zn) exhibits a CFU value of M = 8.52E + 05 (SD = 2.170E + 05) (Fig. 6).

Compared to the positive control, all materials show a significant reduction in viable bacteria compared to the positive control. Ti + PEO shows a reduction of 1.1E-02 log units (98.81% reduction), Ti + PEO(+ Ag) of 3.6E-03 log units (99.40% reduction) and Ti + PEO(+ Zn) achieves a reduction of 3.7E-03 log units (99.64% reduction).

All specimens thus achieved a significant reduction compared to the positive control at the type I error of alpha = 0.05, whereby the real p-values for all comparisons were determined to a Bonferroni-adjusted value of p < 0.001. The two comparisons Ti + PEO vs. Ti + PEO(+ Ag) and Ti + PEO(+ Zn) could also be categorised as significant with p < 0.001. The comparison of Ti + PEO(+ Ag) and Ti + PEO(+ Zn) is classified as non-significant with p = 0.6693.

Overall, the Ag and Zn-loaded implants were able to significantly inhibit bacterial viability and colony formation on the surface of modified Ti implants.

#### Total bacterial DNA count

The negative control showed a mean bacterial count of M = 4.47E + 03 with a standard deviation of SD = 1.647E + 03. For the positive control, the mean bacterial count was M = 3.99E + 09, with a standard deviation of S = 7.149E + 08. For the Ti + PEO specimen, the bacterial count was reduced compared to the positive control, with a mean of M = 7.28E + 04 and a standard deviation of SD = 4.696E + 04. The log-unit reduction was 1.8E − 05, equivalent to a reduction of 99.993%. The Ti + PEO (+ Ag) specimen achieved a mean bacterial count of M = 8.33E + 04, with a standard deviation of SD = 1.253E + 04. This corresponds to a log-unit reduction of 2.1E − 05 and a reduction of 99.998% relative to the positive control. The Ti + PEO (+ Zn) specimen had a mean bacterial count of M = 2.05E + 05 and a standard deviation of SD = 7.604E + 04. The log-unit reduction was 5.1E − 05, representing a reduction of 99.989%.

**Fig. 7:**
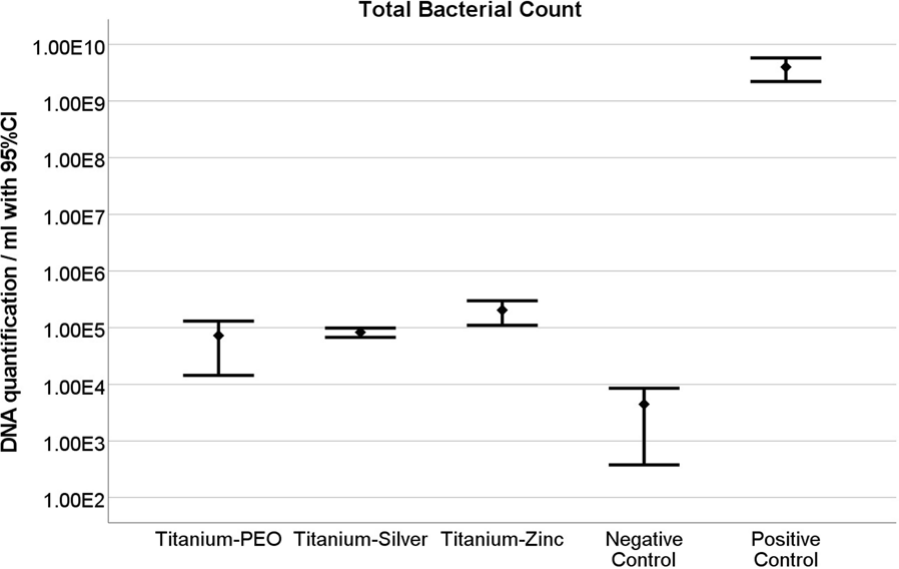
Quantification of mean bacterial DNA copies measured by qrt-PCR (Universal Poly-Primer results) after 48 h for Ti + PEO(+ Ag), Ti + PEO(+ Zn), and Ti + PEO. Mean values with 95% confidence interval are given in logarithmic scale unit. Qrt-PCR results indicate total bacterial DNA count. *N* = 5/implant variant; negative control (*n* = 3); positive control (*n* = 3). *p < 0.05

These results indicate that all tested Ti + PEO modifications significantly reduced the bacterial count compared to the positive control, with Ti + PEO(+ Ag) achieving the highest reduction rate. The bacterial count reductions for all specimens were statistically significant compared to both the positive and negative controls, with p < 0.0001 (Fig. 7). However, the comparison between Ti + PEO and Ti + PEO(+ Ag) was not statistically significant (p = 0.3058), indicating similar antibacterial efficacy. In contrast, Ti + PEO showed a significantly lower bacterial reduction compared to Ti + PEO(+ Zn) (p = 0.0002). Additionally, the difference between Ti + PEO(+ Zn) and Ti + PEO(+ Ag) was also statistically significant (p = 0.0021), reflecting variations in their antibacterial effectiveness. Detailed analysis of specific primers by qrt-PCR confirmed that the dominant bacterial strain on Ti + PEO and Ti + PEO(+ Ag) samples was Fusobacterium nucleatum, while on Ti + PEO(+ Zn) samples Actinomyces naeslundii was detected as the dominant bacterial strain.

## Discussion

The CFU assay in the present study showed that the number of viable bacteria in the specimens loaded with Ag and Zn was significantly lower compared to the unloaded control group. Moreover, significantly lower viable bacterial DNA counts were detected in the Ag and Zn specimens compared to the positive control group (mixed bacterial culture solution).

In summary, biomaterial-associated infections are destructive complications that can occur after the placement of dental implants, called peri-implantitis. The disease often leads to persistent pain and loss of implant function for the patients [45]. In fact, the implantation zone is an accessible target for bacterial attack through colonization and biofilm formation due to the weakened body defense system in the presence of foreign materials. Nowadays, there are several international efforts to address the risk of these infections, however, surgical site infections continue to occur in staggering numbers. According to recent recommendations from many scientific forums, researchers should focus on the developing effective antibacterial surfaces that prevent bacteria from adhering, colonizing, and proliferation of bacteria to local tissues [46]. Therefore, the development of multifunctional dental implants with improved osseointegration and antibacterial activity is considered an advanced approach to the prevention of peri-implantitis [47–49]. Recently, several studies have focused on the development of different surface modifications that have both osseointegrative and antibacterial properties [35, 50–53]. Various techniques have been considered for the production of antibacterial coatings, such as plasma immersion ion implantation (PIII), chemical treatment, anodization, physical vapor deposition (PVD) and plasma electrolytic oxidation (PEO) [54]. The potential of PEO surface treatment was exploited to improve biofunctionality, surface roughness, porosity, and biomechanical fixation [55]. Through this process, the produced porous layer can provide promising osteointegration for implants. However, such porous layers may also provide suitable surfaces for bacterial growth. Accordingly, it has been highly recommended to develop antibacterial PEO coatings that can inhibit bacterial adhesion and biofilm formation and therefore, prove to be ideal for biomedical applications [56]. In this context, this in vitro study examined the effects of Ag and Zn particles in PEO-treated implants on the prevention of peri-implantitis.

For use in dental implants, adequate biocompatibility is essential. PEO surface modification is known to be a safe surface modification process and has shown good biocompatibility results in previous studies [57–59]. During this process, the formation of a thin oxide layer (< 10/µm) on Ti-based implants is enabled by short-lived discrete plasma ignitions that create high temperatures and thus include both, the substrate material as well as species and additives of the electrolyte to participate in the layer formation [60]. This ceramic like porous layer can protect the cells from toxic alloying elements as well as protect the substrate from aggressive and corrosive media. Moreover, according to literature, the microstructure and high surface energy of Ti oxide can stimulate angiogenesis, which contributes to good biocompatibility and the osseointegration process [61]. To assess the biocompatibility of the functionalized Ti implants, a live/dead staining assay was performed on L929 mouse fibroblasts. The results of this assay showed that all implants showed high biocompatibility. Nonetheless, the Ti + PEO(+ Ag) specimens had fewer fibroblast cells on their surface compared to the Ti + PEO(+ Zn) and Ti + PEO groups. Correspondingly, similar results were reported in previous studies showing that Ag ions are associated with oxidative stress and cell detachment in the subjected cells [62, 63]. According to these observations, we concluded that specific concentrations of Ag particulates were associated with higher cytotoxicity. Furthermore, we suspected that this result may be attributed to Ag particualtes producing intracellular reactive oxygen species (ROS) and inhibiting the function of antioxidant enzymes. These processes disrupt normal cell function and lead to cell membrane breakdown, inducing apoptosis in cells [64]. In addition, Ag particulates can attach to functional groups of cell proteins, resulting in physicochemical interactions within cells [65]. At concentrations less than 25 mg/L, most studies show that Ag micro- and nanoparticulates are harmless and not cytotoxic [63, 66, 67]. Nonetheless, the cytotoxicity of Ag particulates may be inversely related to their size, with larger particles exhibiting lower cytotoxicity due to a lower surface-to-volume ratio [63, 68–71].

The main objective of this study was to induce PEO modification of Ti implants to antibacterial properties. For this purpose, Ag and Zn were incorporated onto the surface of dental implants and the antibacterial potential was investigated using CFU and qrt-PCR analysis. Considering the knowledge of specific bacterial variations within the oral cavity, *Streptococcus mutans*, *Actinomyces naeslundii*, *Fusobacterium nucleatum* and *Porphyromonas gingivalis* were selected as common oral microbes for the preparation of the bacterial mixture. After incubating the Ti + PEO(+ Ag) and Ti + PEO(+ Zn) implants in the bacterial mixture, the bacterial load on the specimen surface was expected to be minimized. The results of the CFU assay showed a significant reduction in colony formation for the Ti + PEO(+ Ag) and Ti + PEO(+ Zn) specimens compared to the non-functionalized (Ti + PEO) and the positive control group (mixed bacterial culture solution). Similar results were reported for Souter’s et al. 2020 [72] in vitro experiment in which Ag ions were incorporated into Ti orthopedic implants and the bacterial content on the surface of the implants was examined through CFU assay. They found that significantly fewer bacteria colonized the Ag-coated implants than the untreated control group after 24 h. Further, Masamoto et al. reported similar results for Ag ion coated cp-Ti (non-alloyed Ti with a purity of 99.0%—99.4%) implants using CFU measurement [73]. The antibacterial potential of Ag ions is based on interference with bacterial DNA replication and inactivating of cellular protein production [74]. The ions bind to the functional groups of proteins, leading to protein denaturation [75]. Together with activating the production of reactive oxygen species (ROS), all these processes lead to a significant reduction of bacterial colonization on implants coated with Ag ions [76]. Similarly, Zn ions interrupt intracellular activities such as glycolysis, transmembrane proton translocation, and acid tolerance, killing bacteria [77]. It has also been suggested that Zn ions destroy bacterial cells by enhancing oxidative stress in bacterial cells [78].

In this study, qrt-PCR results reflect a lower bacterial DNA count adhering to the surface of all tested specimens compared to the positive control group (mixed bacterial culture solution). Even though there is no significant difference between the Ti + PEO(+ Ag) and (Ti + PEO) groups, there is a significantly higher bacterial DNA count compared to Ti + PEO(+ Zn). This observation was attributed to the porous structure of the oxide layer on the PEO-treated implants. Indeed, this porous layer provides a better basis for cell attachment and osteointegration as the bone grows into the pores [79]. However, it has been shown that increased surface roughness was associated with an increased risk of bacterial attachment [80]. Accordingly, Van Hengel et al. loaded Ti implants with Ag and Zn ions following PEO surface modification and reported a higher antibacterial effect of Ag implants compared to Zn implants [6]. A possible explanation for this discrepancy lies in the concept of minimum inhibitory concentration (MIC) for Ag and Zn ions. It has been suggested that this difference is caused by the MIC of Zn ions being 100 to 150 times higher than that of Ag ions [81]. This means that the lowest concentration of Zn ions required is significantly higher than the lowest concentration of Ag ions required to inhibit the visible growth of bacteria after overnight incubation. In conclusion, in this study, PEO-treated Ti implants with antibacterial activity were successfully fabricated and tested in vitro as potential approach to counter implant-related infections such as peri-implantitis caused by common oral bacteria. Our results show promising biocompatibility and antibacterial activity of Ag and Zn-loaded PEO-Ti samples compared to non-functionalized Ti samples. Further studies with a modified experimental setup, e.g., with an adjustment of parameters of the PEO process, should be performed to further optimize the developed antibacterial implants.

Despite these advances, our study shows limitations. As described in the Methods section, the solid implant specimens were incubated with a mixed bacterial culture and subsequently vortexed in PBS. Ideally, the extracts would represent the antibacterial effect and show an exact measure of viable or dead bacteria after treatment with Ag or Zn ions. However, the exact measure of antibacterial activity could not be confirmed experimentally as the test extract inevitably contained some amount of loosely attached viable or dead bacterial cells. Further, the cultivation of the bacterial strains was challenged due to the individual time-dependent growth rates of the respective strains, thus the amount of each bacterial colony within the mixed culture solution may have varied. Finally, other factors that may influence the efficiency of the antibacterial effect, such as the in vivo retention of Ag and Zn ions and their impact on the duration of antibacterial activity, could not be accounted for in this setting.

## Conclusion

To improve the durability of dental implants, multifunctional implant surfaces have been developed to both inhibit peri-implantitis and promote osteointegration of the implant into the host bone tissue. PEO surface modification was used to incorporate Ag and Zn ions as antibacterial agents onto the surface of Ti implants by adding appropriate chemical compounds to the electrolyte used. The implants loaded with metal ions eradicated the formation of bacterial colonies after 48 h of incubation in a bacteria mixture. In addition, the biofunctionalized Ag and Zn implants represented a significantly lower bacteria DNA count. Therefore, implants biofunctionalized with Ag and Zn particulates by means of PEO hold great potential for further development towards multifunctional dental implants. Our study demonstrated that Zn-loaded titanium specimens exhibited significantly lower cytotoxicity compared to Ag-loaded counterparts while maintaining similar antibacterial efficacy. Although Ag coatings show strong antibacterial effects, their potential to hinder osseointegration raises concerns, making Zn-loaded specimens a more favorable option for clinical applications.

## Data Availability

No datasets were generated or analysed during the current study.

## Abbreviations

PEO: Plasma-electrolytic oxidation
Zn: Zinc
Ag: Silver
Ti: Titanium
SEM: Scanning electron microscopy
PBS: Phosphate-buffered saline
DNA: Deoxyribonucleic acid
qrt-PCR: Quantitative real-time polymerase chain reaction
CFU: Colony forming unit
EDS: Energy dispersive X-ray spectroscopy
SE: Secondary electron
PI: Propidium iodide
FDA: Fluorescein diacetate
PII: Plasma immersion ion implantation
PVD: Physical vapor deposition
MIC: Minimum inhibitory concentration
